# Impact of a Banning Indoor Dining Policy on Restaurant Avoidance Behavior during the COVID-19 Outbreak

**DOI:** 10.3390/ijerph18147268

**Published:** 2021-07-07

**Authors:** Tai-Hsiang Chen, Lan-Lung (Luke) Chiang, Chen-Chung Ma, Chiu-Hua Chang

**Affiliations:** 1 College of Management, Yuan Ze University, Taoyuan 32003, Taiwan; scts1215@yahoo.com.tw (T.-H.C.); lukech@saturn.yzu.edu.tw (L.-L.C.); 2 Department of Healthcare Administration, I-Shou University, Kaohsiung 82445, Taiwan; 3 Nursing Department, Kaohsiung Veterans General Hospital, Kaohsiung 813414, Taiwan

**Keywords:** coronavirus disease 2019, theory of planned behavior, consumer behavior, partial least squares

## Abstract

The coronavirus disease 2019 (COVID-19) is spreading around the world, and Taiwan is no exception. Faced with the outbreak of the epidemic, the Taiwan government immediately ordered a policy banning indoor dining. The main purpose of the present research is to extend a Theory of Planned Behavior (TPB) theoretical framework to explore the public perception toward banning indoor dining policy on restaurant avoidance behavior during the COVID-19 outbreak. An online survey was administered in Taiwan during the COVID-19 pandemic from 25 May to 8 June 2021; a total of 326 responses were collected by a convenience sampling method, and partial least square (PLS) analysis was deployed to examine the hypothesized relationships. The results showed that perception toward banning indoor dining policy had independent significant associations with attitude, perceived behavioral control, and restaurant avoidance behavior. Moreover, attitude, perceived behavioral control, and subjective norm had independent significant associations with restaurant avoidance behavior. This study provides theoretical and practical insights into the psychological and behavioral processes involved in policy by the general public during the COVID-19 pandemic, thus helping policymakers to better understand public opinion and responses to policy issues.

## 1. Introduction

The global pandemic caused by the coronavirus disease 2019 (COVID-19) has so far had a significant impact on human life and health. Due to the rapid spread of the epidemic, countries around the world have adopted border quarantine and post-entry control measures. Notably, the spread of COVID-19 has led to a sudden and temporary sharp drop in revenue for companies in many industries, the most severe being the hospitality industry. Since more than 100 countries declared partial or total blockades, air and intercity travel in major cities around the world is down 70–90 percent from the previous year [1]. As a result, international, regional, and local travel restrictions have greatly harmed the hospitality industry because of its dependence on human mobility [2]. In the context of the hospitality industry, the restaurant industry is considered to have extensive and high-level business risks [3], which have been seriously aggravated during the pandemic. In fact, several professional reports have concluded that COVID-19 has had a serious impact on the restaurant industry as the epidemic has raged on. For example, since the end of March 2020, 3% of restaurants have permanently closed due to COVID-19, and the restaurant industry may have lost nearly $120 billion in sales in the first three months of the COVID-19 pandemic [4].

The restaurant and food service sectors have been amongst the worst affected, with severe losses to sales and jobs. Given that restaurant customers are anxious about and concerned with safety issues throughout the food consumption process [5], they may call for different types of contactless services from restaurants, which are essential to contain the spread of infections. A similar situation has been observed in Taiwan. At the same time as the outbreak, Taiwan quickly set up the Central Epidemic Command Center (CECC) to coordinate the situation of the epidemic. The CECC not only fully integrates government resources, coordinates the local governments of various counties and cities, but also strengthens the legal basis for various administrative operations. Although the CECC immediately planned and launched the epidemic control measures, the Ministry of Economic Affairs reported that April 2020 was the worst month on record for food and beverage sales. There was a 22.8% year-on-year decline for the sector, which was equivalent to USD 1.6 billion and the steepest drop ever recorded [6]. After more than a year of efforts, COVID-19 has officially invaded the Taiwanese community. In order to avoid more serious infection in the community and impact on the medical system and social daily life, all counties and cities in Taiwan have banned indoor dining since May 20. However, this will bring greater losses to the restaurant industry, but it is necessary to reduce the risk of infection in the community. Moreover, COVID-19 is transmitted mainly through droplets and contact, and thus dining in close proximity may increase the risk of developing the disease [7]. Given the lockdowns, social distancing measures, and general caution towards places where people congregate, it is worth discussing whether the public perception toward the epidemic prevention measures can be coordinated with the government’s efforts to prevent dining in restaurants.

Accordingly, the conceptual framework from the present research is derived from the theory of planned behavior (TPB) [8,9]. The TPB [8,10] is one of the most important theories used to predict individual behavior. The TPB posits that the willingness of individuals to execute a certain behavior is affected by attitude, subjective norm, and perceived behavioral control. These types of volitional and nonvolitional dimensions are the key components of the TPB [11,12]. The theoretical significance of the previous study is an important part of verifying the TPB theory and helps to better understand the consumption patterns of diners [13]. In addition, despite the high applicability of TPB theory, few empirical studies have extended TPB theory to public behavior in the context of the epidemic, even in Taiwanese society, which has recently been severely affected by COVID-19. The main purpose of the present research is to extend a TPB theoretical framework to explore the public perception toward banning indoor dining policy on restaurant avoidance behavior during the COVID-19 outbreak. In the sense of risk awareness, people recognize the risk of infection when they go out, so they choose to cook by themselves without going out for dinner and avoid the decision-making behavior of going to the restaurant. Not to mention how citizens should respond to government decisions and policies in the face of a pandemic. When positing a perception toward government policy, trust is considered to be a substantial determinant of perception and reactions to some potential risks [14]. Trust in government refers to trust that it has plenty of control over a particular, serious event and is held responsible for public protection. Undoubtedly, the international restaurant industry cannot be the same as before after the pandemic, even if COVID-19 is completely under control. It is now more important than ever before for the restaurant industry and even the government to understand the decision-making of dining behavior. Consequently, a multivariate data assessment and process are established to achieve the study objectives.

## 2. Literature Review

### 2.1. The Theory of Planned Behavior

The TPB is one of the most widely used theories of social psychology, which aims to predict human decision-making and behavior [11,15]. Its applicability and predictive power for different human behaviors have been demonstrated through meta-analyses [16]. The TPB proposes three antecedent variables of behavioral intention: attitude, subjective norm, and perceived behavioral control. Moreover, the TPB has been widely used in the literature to explain behavioral intention [17,18].

Attitude refers to a person’s positive or negative feelings about a particular behavior [19]. That is to say, attitude evaluates whether an individual’s behavior is beneficial to him/her or whether the individual likes it. When individuals have a more positive attitude, their behavioral intentions are also higher. On the contrary, the more negative an individual’s attitude is, the lower his behavioral intention will be.

Subjective norm is a kind of social factor that refers to whether social pressure influences individuals to carry out a specific behavior [19]. In some cases, individuals’ behavior is influenced by external social pressures, mainly from people they trust to be important, such as peers, family, health care providers, and supervisors.

Perceived behavior control refers to the possible interference or obstacle perceived by the individual according to their past experience when carrying out a specific behavior [10]. When individuals have more resources and more opportunities (i.e., higher perceived behavioral control), they are more willing to engage in specific behaviors.

A previous study suggested that using only a single belief, such as an attitude, subjective norms, and perceived behavioral control, can limit our understanding of behavioral intention in the TPB [20]. Therefore, we still need to further expand the scope of the TPB, for example, to include other variables, in order to enhance our understanding of the issue [20].

### 2.2. Perception toward Banning Indoor Dining Policy

During the first wave of the pandemic in mid-March 2020, most restaurants were mandated to suspend dine-in services, and only takeout, drive-thru, or delivery services were permitted [21]. The fact that the United States planned this early in the outbreak shows that it was an important policy decision. Lawmakers in many of the nation’s most populated counties announced some of the most drastic measures to enforce social distancing by ordering bars and restaurants to close their dining rooms for extended periods of time. Countries all over the world are also imitating this epidemic prevention model [22]. While these intervention efforts have minimized personal interaction and alleviated the virus’s spread, they have greatly threatened the restaurant industry’s survival.

The CECC in Taiwan raised the alert in Taipei City and New Taipei City to Level 3 from 15 May to 28 May [23]. Everyone must wear a mask outdoors and avoid unnecessary movement, activities, or gatherings. All family or social gatherings involving over five people indoors or 10 people outdoors are suspended. In addition, food and beverage vendors should use epidemic prevention measures, such as contact-information registration, social distancing, and dividers. Those that cannot adopt those measures are urged to offer take-out services. However, on 19 May, in view of the epidemic still being severe, the CECC decided that all counties and cities are upgraded to Level 3 of epidemic prevention [24]. As the epidemic continued to heat up, on 20 May, in order to avoid unnecessary clusters and increase the number of confirmed cases of COVID-19, counties and cities also issued directives to ban indoor dining in the restaurant industry in order to reduce the risk of the spread of the epidemic. If that was not possible, the restaurant could choose to close temporarily. Restaurants that violate the rules and refuse to improve were to be fined according to the law [25]. Even supermarkets and convenience stores have banned dining. It was originally scheduled to lift the Level 3 alert on 28 May, but the trend of the epidemic did not decrease, so the CECC announced that the Level 3 alert in all of Taiwan was extended to 12 July [26].

In the face of the threat of COVID-19, everyone has their own values about the no-indoor dining policy, based on their attitude and the diversity of society. The important factors consisting of personal values affect public behavior and opinion about government engagement, and the demand for government involvement, including government regulation, was substantially higher for persons with a high level of concern for the environment and for persons with a high level of perceived consumer effectiveness [27]. In other words, individual values influence opinions about government intervention through regulation and policy management. At the same time, the hypothesis of the influence of personal opinions on the management of banning indoor dining policy can be obtained, that is, the influence of policy perception on acceptance. Therefore, the perception toward government policy is especially affected by trust in the policy-making process, policy consistency, and policy efficiency.

### 2.3. Restaurant Avoidance Behavior

COVID-19 has substantially changed normal life conditions and created a “new normal” that has forced economic and socio-behavioral changes. While pursuing the new normal in people’s way of life, an increasing tendency has been observed to reduce the spread of COVID-19, including handwashing and social distancing as preventative measures, which may be viewed as coping strategies in health promotion and disease prevention. COVID-19 is transmitted mainly through droplets and contact, and social distributive measures are mainly adopted in countries where the epidemic is well-controlled [7]. Persuading the public to observe particular behaviors has been proposed as a means of preventing infectious diseases [28]. Providing safe consumption spaces is a potential evidence-based intervention for reducing COVID-19 infections because most people now avoid consuming dine-in services, which involve physical contact with people. If the restaurant sector is to provide customers with safe consumption spaces, it is necessary to follow prevailing COVID-19 prevention measures. Given the anxiety and concern of restaurant customers about the safety of their food consumption [5], they may request different types of non-contact service, which is critical to controlling the spread of infection.

The restaurant industry across Taiwan has largely followed and obeyed the proposed prevention measures. On 20 May 2021, all counties and cities in Taiwan announced that restaurants would be banned from indoor dining internally. Although it may be inconvenient for the public, at the level of societal change, the public broadly accepts that implementing such behavioral changes can instigate healthier lifestyles. Preventive health behaviors are greatly influenced by perceived risk [29]. Under the risk awareness, the self-cognition and attitude of the public will produce the decision behavior of avoiding going to the restaurant. Restaurant avoidance behavior represents a potential psychological role in promoting physical and mental health and addressing social decorum and etiquette, which means that the consumers may show extreme reluctance behavior to dine out.

## 3. Methodology

### 3.1. Conceptual Framework and Research Hypotheses

**Figure 1:**
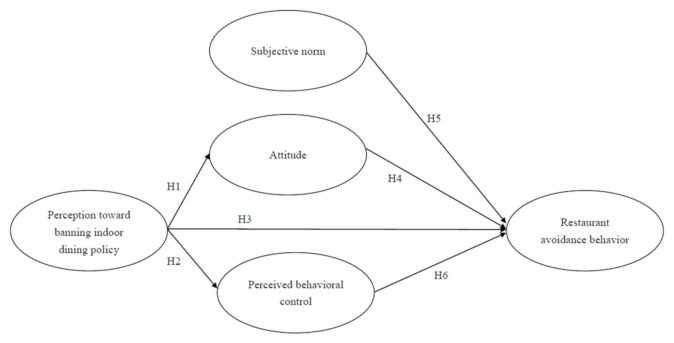
Research framework.

Our study adopted the TPB as the theoretical basis to investigate the avoidance behavior of dining in restaurants during a pandemic. As Figure 1 shows, the three primary determinants jointly influence the intention to avoid dining in restaurants according to the TPB [10]. Furthermore, our study extended the TPB by including a construct of the perception of policies banning indoor dining. In particular, the subjective norm of TPB theory refers to an individual’s perception of whether a significant other thinks he or she should participate in the activity. Thus, subjective norms and perceptions toward banning indoor dining policy belong to psychic perception, but at different levels and groups. In the pretest process of our study, experts and scholars pointed out that the difference between the two may make the respondents confused. For the above reasons, this study does not examine the relationship between the two. This study considered restricting the public diet to reduce the risk of community transmission of the disease. Noting that encouragement for sanitation and hygiene-related behaviors has contained previous infectious diseases, individuals can be easily educated about COVID-19 prevention measures or policies, thereby reducing its wider impacts and bringing positive benefits [30]. Understanding preventive behaviors and risk perceptions towards COVID-19 is crucial for practicing disease prevention and enhancing risk awareness in the restaurant sector. Based on the extant literature reviewed earlier, the following hypotheses are proposed.

**Hypothesis** **H1:** *There is a positive relationship between perception toward banning indoor dining policy and attitude*.

**Hypothesis** **H2:** *There is a positive relationship between perception toward banning indoor dining policy and perceived behavioral control*.

**Hypothesis** **H3:** *There is a positive relationship between perception toward banning indoor dining policy and restaurant avoidance behavior.*

**Hypothesis** **H4:** *There is a positive relationship between attitude and restaurant avoidance behavior*.

**Hypothesis** **H5:** *There is a positive relationship between subjective norm and restaurant avoidance behavior*.

**Hypothesis** **H6:** *There is a positive relationship between perceived behavioral control and restaurant avoidance behavior*.

### 3.2. Sampling and Data Collection

The hypothesis model was tested by quantitative investigation design. In the context of the current COVID-19 pandemic, it was appropriate to use online questionnaires and surveys to solicit responses in order to limit face-to-face contact and comply with prevailing social distance requirements. This prompted us to collect online data using SurveyCake, a trustworthy and world-class cloud-based survey service to create professional online customer surveys. The online survey of our study was open to the public in Taiwan from 25 May to 8 June 2021. A total of 326 responses were collected by a convenience sampling method. Participants were given a brief statement (on a page before the survey began) about the purpose of the study, the method of data collection, and information about the legal requirements for data protection. On the last page, the researchers attached a short statement stating that “clicking the final ‘Submit’ button signifies that the respondent agrees to participate in the study.” The data analysis of this study was limited to participants over the age of 20 who normally had the habit or experience of dining in restaurants and whose eating behaviors were affected by the COVID-19 outbreak. Online surveys were typically completed in less than 10 min. Due to the observational nature of the study, the present study did not require Institutional Review Board approval of the local Ethics Committee. Nonetheless, participants were fully informed about the study participated on a voluntary basis, and the survey responses were anonymous.

### 3.3. Measures

The survey instrument comprised three sections and was based on items drawn from the literature. The first section included a description of the research. The second section of the questionnaire collected demographics such as gender, age, marriage, education, and occupation. The third consisted of five constructs related to the hypotheses. The attitude, subjective norm, and the perceived behavioral control concept contained in TPB theory were adapted from prior research [31]. In particular, four items for the attitude, three items for the subjective norm, and two items for the perceived behavioral control were used. Respondents were asked to indicate what they believed to be the underlying psychological factors of dining in the restaurants during COVID-19 in order to predict and understand the behavior of the general public. The three items used to measure perception toward banning indoor dining policy were adapted from a study by Kang and Park [32]. Respondents were asked to indicate how much they trusted government policies. The restaurant avoidance behavior concept was adapted with three items drawn from Zhong et al. [22]. Respondents were asked to indicate their risk perception and psychological decision-making behavior when dining in restaurants under the threat of COVID-19. The questionnaire items were mostly adapted from previous literature and slightly modified to fit the context of this study, and all items were assessed on a seven-point Likert-type scale (from 1 = strongly disagree to 7 = strongly agree). The survey questionnaire containing these measures was pretested with experts and academics. A slight amendment was made based on their feedback.

### 3.4. Analysis

The collected data were analyzed by SPSS 25 statistical software, and we used partial least square (PLS) technique, supported by SmartPLS 2.0 M3 software [33], to validate the proposed model in two stages: measurement model and structural model [34].

## 4. Results

As shown in Table 1, most respondents in the sample were female (65.6%, *n* = 214), between the ages of 30–39 (37.4%, *n* = 122), and were married (61.7%, *n* = 201). Most respondents had a college education (51.2%, *n* = 167) and were employed in the private enterprise (50.6%, *n* = 165).

### 4.1. Measurement Model Evaluation

As suggested by Hair et al. [34], this study utilized individual question factor loading, composite reliability (CR), average variance extraction (AVE), and Cronbach’s α to assess reliability. We compiled the results in Table 2. First, according to Hair et al. [34], the factor loading must be higher than the standard 0.75. The results of the analysis showed that the factor loading of each question for attitude, subjective norm, perceived behavioral control, perception toward banning indoor dining policy, and restaurant avoidance behavior was higher than the standard 0.75. In addition, the CR and Cronbach’s α should be higher than the standard 0.7, and the AVE should be higher than the standard 0.5 [34]. The results showed that the CR and Cronbach’s α were higher than 0.9 and the AVE was higher than 0.7, all of which were above the standard, indicating that the constructs and measurement variables of this study had sufficient reliability.

Furthermore, the validity was assessed by convergent validity and discriminant validity, and the results are shown in Table 2. The AVE was used to interpret and measure the extent of potential variables, as suggested by Fornell and Larcker [35]. The analysis of this study showed that the AVE was higher than 0.7 for all components. The criterion being higher than 0.5 indicates adequate convergent validity. The discriminant validity was attained if the square root of AVE for each construct is higher than the correlations between the construct and the other constructs [35]. As shown in Table 3, the square root of AVE for each construct was between 0.85 and 0.95, which was higher than the correlation coefficient between the constructs. Then, it could be inferred that the theoretical model had sufficient discriminant validity. After the above, the study had sufficient reliability and validity to proceed to the next step of structural model analysis.

### 4.2. Structural Model Evaluation

**Figure 2:**
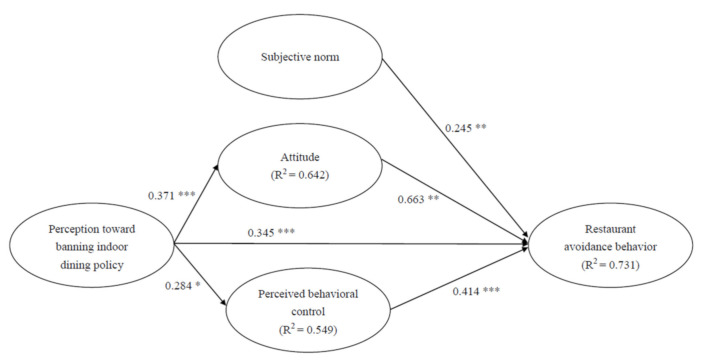
Path Coefficient Analysis * *p* < 0.05, ** *p* < 0.01, *** *p* < 0.001.

The structural model evaluated the path coefficients between constructs based on directionality and significant correlation. A bootstrapping procedure was used to test the statistical significance of each path coefficient. The results of the Smart-PLS part coefficients and significance values are shown in Figure 2. Table 4 shows the summary of our hypothesis testing. All of the proposed hypotheses were supported. Attitude was significantly influenced by perception toward banning indoor dining policy (β = 0.371, *p* < 0.001), providing support for H1. Perceived behavioral control was significantly influenced by perception toward banning indoor dining policy (β = 0.284, *p* < 0.05), providing support for H2. Restaurant avoidance behavior was significantly influenced by perception toward banning indoor dining policy (β = 0.345, *p* < 0.001), attitude (β = 0.663, *p* < 0.01), subjective norm (β = 0.245, *p* < 0.01), and perceived behavioral control (β = 0.414, *p* < 0.001), providing support for H3, H4, H5, and H6. Overall, the model explained about 64.2%, 54.9%, and 73.1% of the determined variance in the attitude, perceived behavioral control, and restaurant avoidance behavior, respectively.

## 5. Discussion

In the global COVID-19 outbreak, Taiwan was also threatened by the epidemic after more than one year of prevention, which had a significant impact on the whole social economy, especially the restaurant industry. Therefore, our theoretical framework was built on the TPB and focused on the restaurant avoidance behavior under the policy of prohibition of indoor dining. It is worth noting that this study was conducted in Taiwan during a pandemic, where the government and local residents learned from the 2002 SARS outbreak and responded faster and more effectively. Therefore, this study collected the insights of Taiwanese public under special circumstances and produced meaningful findings.

First, according to the results of this empirical study, the perception toward banning indoor dining policy is positively associated with attitude and perceived behavioral control. Perceived behavior control reflects the amount of resources and opportunities an individual has to engage in the behavior, while attitude reflects the positive or negative evaluation of the policy held by the individual. This study points out that the degree of personal perception of policy further influences the generation of behavior due to subjective psychological feelings. Since the statistical results of the PLS estimates make it possible to estimate the causal relationship between these two variables; this corresponds to previous studies, which have shown that a good attitude towards government policies is conducive to the adoption of policies [36] and effective implementation [37]. Understanding public attitudes toward policies and regulations can help gauge public acceptance and likely response to those policies. Zhang et al. [38] believe that residents are more likely to act in a friendly way with behavioral control because the national government implements policies out of reasonableness. Specifically, the more that the public becomes aware of the policy in detail and senses the difference in general life that the policy seeks to impose, the more customers consider that public opinion is sufficiently regarded in the course of establishing the policy. The public is aware of the risk of infection when they go out, so they can have appropriate behavioral control to avoid the decision-making behavior of dining in restaurants. In addition, they believe that a legitimate policy review has been conducted and they believe that even if there is any change in political power as a result of the COVID-19 outbreak, the policy will be maintained. Similarly, our study supports the fact that the perception toward banning indoor dining policy is positively associated with restaurant avoidance behavior. In the face of the sudden COVID-19 epidemic, a sound country should have an emergency management system and quickly strengthen the institutional construction of laws and policies. Only when the public has confidence in the government can the prevention and control of the epidemic be successful and effective [39]. Zhao and Zhong [40] found that government propaganda or policies have a positive impact on individuals’ willingness. A crisis is a time to test the capability and style of the government, but also an important opportunity to build public satisfaction and trust in the government. Efficient government decision making and proper handling will rapidly enhance the relationship of trust between the government and the public. On the contrary, it will develop into distrust of the government and further damage the credibility of the government. A past study proposed that government policies can significantly influence public behavior [41]. During the outbreak of community infection in Taiwan, the government of each county and city quickly implemented the policy of banning domestic consumption. The public recognized the risk of infection when dining out and chose to believe the government’s propaganda, which eventually resulted in restaurant avoidance behavior. Therefore, the government should not only respond to the epidemic in a positive and effective way but also shape the personality charm of the leaders and show the ability and wisdom of the government to serve the people.

Moreover, the present study supported the fact that the perception toward banning indoor dining policy is positively associated with restaurant avoidance behavior, which matches the findings observed in a prior study [42]. Based on the findings, this study points out that perceived effectiveness of policies increases the attractiveness of behaviors, meaning the extent to which the public recognizes and believes that policies play a role in solving problems or motivating behaviors. Because blocking the spread of the epidemic is considered to be an important issue, the perception of more effective policies can promote higher adoption rates of sustainable behaviors. The previous study has used TPB theory to explore behaviors related to food waste and intentions to avoid it and suggests that government policies should be more diverse to better address specific recommendations for each link [43]. Liu et al. [44] emphasized that the government has sufficient power and resources to promote various activities to cultivate public awareness of environmental issues and that the promotion of policies has a strong predictive effect on public support and behavioral willingness. High-level policy awareness helps to translate awareness into action. Therefore, it is necessary for the public to understand why the government has put forward the policy of banning indoor dining and raise the level of awareness so as to stimulate the generation of behavior. The results also have implications for public attitudes and behavior control. A prior study on infectious disease epidemics showed that attitude, awareness, and risk perception help motivate people to adopt preventive behaviors [45]. In addition, similar to the present study, the related constructs of TPB were positively associated with behavior, which can help promote and maintain public preventive behavior in the context of the COVID-19 pandemic. Perceived behavioral control, attitudes, and subjective norm are closely related to each other. Pandey et al. [46] have extended TPB theory to focus on food consumers’ attitudes and even perceived behavioral control so as to understand consumers’ behavioral intentions. There is evidence that the three constructs of TPB theory are important predictors of preventative behavior [47]. If the public is to take preventive action after receiving information, they need to believe that the action is effective. This result also supports previous studies [48,49], which indicated the importance of TPB in explaining safe/risky behavior. This finding provided us with valuable information that individuals’ psychological perception is critical when they make any decisions related to safe behavior choices or risk-taking. When the epidemic threatened people, everyone thought in the same way. All people must face the virus together and cooperate with the government’s policy of banning indoor dining. The results of this study also support this view. Thus, in the context of the COVID-19 pandemic, each component is likely to be a key determinant in predicting whether or not people adopt preventive behaviors. For example, people may be more willing to use hand sanitizers if they think they are easy to use (high perceived behavioral control) and effective (positive attitudes toward hand sanitizers) and that everyone else does it (high subjective norm). Understanding how the three core components of the TPB have worked during the COVID-19 pandemic could provide valuable insights to public health organizations to accelerate the effectiveness of policy implementation during this pandemic and future outbreaks.

This study contains some limitations that provide opportunities for future research. First, we used an online questionnaire to collect data, so people who may have limited Internet access are not included in this sample. Second, our study did not broadly explore other factors associated with COVID-19 behavior, such as those that might influence public knowledge. It is suggested that future research could extend the framework of this study to achieve an effective two-way interaction between the public and the government. In addition, since the study was conducted during a sudden and unstable pandemic, it may lack the depth of a longitudinal study. Finally, the possible relationship between perceived and actual risks over time should be taken into account, particularly in terms of the degree of containment of the epidemic over time.

## 6. Conclusions

During health crises and emergencies, the public needs to be ready to take precautions, as the novelty and unpredictability of outbreaks can largely overwhelm the capacity of health systems. The main purpose of the present study is to explore the public perception toward banning indoor dining policy on restaurant avoidance behavior during the COVID-19 outbreak. This study provides evidence that can help policy makers to better understand public perceptions of these policy issues and provides critical and timely insights into how governments can establish and implement appropriate policies and interventions. The government has proposed a ban on indoor dining to prevent the spread of the disease in communities. Perception of policy helps to translate knowledge into action. The spirit of mutual trust, cooperation, and shared risk formed among people, society, and government in times of crisis is the foundation necessary to build a strong social support system to overcome the crisis. This study encourages the general public to adhere to the banning indoor dining policy during the COVID-19 pandemic and continues to guide them towards a positive attitude towards the policy.

## Figures and Tables

**Table 1:** Demographic information of respondents.

Demographic Information	Categories	Cases	Percentage
Gender	Female	214	65.6%
	Male	112	34.4%
Age	20~29	82	25.2%
	30~39	122	37.4%
	40~49	56	17.2%
	50~59	37	11.3%
	≥60	14	4.3%
Marriage	Married	201	61.7%
	Unmarried	114	35.0%
	Other (including divorced, widowed, separated, etc.)	11	3.3%
Education	Master or above	54	16.6%
	College	167	51.2%
	Junior college	41	12.6%
	High School	58	17.8%
	Junior high school or below	6	1.8%
Occupation	Public Sector	43	13.2%
	Private Enterprise	165	50.6%
	Freelance	63	19.2%
	Self-employment	27	8.1%
	Home Management	14	4.3%
	Student	15	4.6%

**Table 2:** Reliability and validity analysis.

Constructs/Items	Factor Loading	AVE	CR	Cronbach α
Attitude		0.841	0.936	0.923
Not to dine in the restaurant would be reassuring.	0.796			
Not to dine in the restaurant would be safe.	0.778			
Not to dine in the restaurant would be pleasant.	0.803			
Not to dine in the restaurant would be worthwhile.	0.901			
Subjective norm		0.796	0.938	0.925
My friends think I should not dine in the restaurant.	0.891			
My families think I should not dine in the restaurant.	0.902			
Most people I consider important think I should not dine in the restaurant.	0.856			
Perceived behavioral control		0.727	0.925	0.916
Not to dine in the restaurant would be easy for me.	0.814			
I’m sure I’ll not dine in the restaurant, and this decision is entirely up to me.	0.851			
Perception toward banning indoor dining policy		0.924	0.914	0.907
I think that the policy could be managed continuously in spite of the turnover of political power.	0.867			
I think that public opinion was sufficiently regarded when the policy was established.	0.933			
I think that the project evaluation related to the policy is trustworthy and legitimate.	0.842			
Restaurant avoidance behavior		0.892	0.948	0.912
I feel worried about dining in the restaurant.	0.902			
Dining in the restaurant may not be safe for me.	0.861			
Dining in the restaurant may cause me to get infected with COVID-19.	0.792			

Note: AVE = average variance extracted; CR = composite reliability.

**Table 3:** Discriminant validity analysis.

Variables	1	2	3	4	5
Attitude	0.868				
Subjective norm	0.767	0.919			
Perceived behavioral control	0.701	0.843	0.894		
Perception toward banning indoor dining policy	0.694	0.732	0.817	0.941	
Restaurant avoidance behavior	0.794	0.801	0.786	0.652	0.923

**Table 4:** Research hypothesis validation.

Hypothesis	Path Coefficient	t-Value	Results
H1 Perception toward banning indoor dining policy → Attitude	0.371 ***	9.41	Supported
H2 Perception toward banning indoor dining policy → Perceived behavioral control	0.284 *	3.62	Supported
H3 Perception toward banning indoor dining policy → Restaurant avoidance behavior	0.345 ***	5.79	Supported
H4 Attitude → Restaurant avoidance behavior	0.663 **	6.51	Supported
H5 Subjective norm → Restaurant avoidance behavior	0.245 **	5.27	Supported
H6 Perceived behavioral control → Restaurant avoidance behavior	0.414 ***	2.59	Supported

* *p* < 0.05, ** *p* < 0.01, *** *p* < 0.001.
